# Extubation Readiness in Preterm Infants: Evaluating the Role of Monitoring Intermittent Hypoxemia

**DOI:** 10.3390/children8030237

**Published:** 2021-03-18

**Authors:** Elie G. Abu Jawdeh, Amrita Pant, Aayush Gabrani, M. Douglas Cunningham, Thomas M. Raffay, Philip M. Westgate

**Affiliations:** 1 Division of Neonatology, Department of Pediatrics, College of Medicine, University of Kentucky, Lexington, KY 40508, USA; doug.cunningham@uky.edu; 2 Department of Pediatrics, McLaren Regional Medical Center, Flint, MI 48532, USA; amrita.pant@mclaren.org; 3 Division of Pediatric Gastroenterology, Hepatology and Nutrition, Department of Pediatrics, University of Texas Southwestern, Dallas, TX 75390, USA; aayush.gabrani@utsouthwestern.edu; 4 Division of Neonatology, Department of Pediatrics, College of Medicine, Case Western Reserve University, Cleveland, OH 44106, USA; tmr12@case.edu; 5 Department of Biostatistics, College of Public Health, University of Kentucky, Lexington, KY 40536, USA; philip.westgate@uky.edu

**Keywords:** extubation, intubation, intermittent hypoxemia, preterm, respiratory distress

## Abstract

Preterm infants with respiratory distress may require mechanical ventilation which is associated with increased pulmonary morbidities. Prompt and successful extubation to noninvasive support is a pressing goal. In this communication, we show original data that increased recurring intermittent hypoxemia (IH, oxygen saturation <80%) may be associated with extubation failure at 72 h in a cohort of neonates <30 weeks gestational age. Current-generation bedside high-resolution pulse oximeters provide saturation profiles that may be of use in identifying extubation readiness and failure. A larger prospective study that utilizes intermittent hypoxemia as an adjunct predictor for extubation readiness is warranted.

## 1. Introduction

Mechanical ventilation is common in preterm infants in the treatment of respiratory distress and respiratory failure [1]. Although essential and lifesaving, prolonged mechanical ventilation is associated with increased morbidities [2]. Nevertheless, untimely extubation may also be harmful, as failure and subsequent reintubation is associated with increased morbidity and mortality as well [3,4,5]. Therefore, it is imperative that a timely and safe extubation be undertaken to shorten the duration of mechanical ventilation after the resolution of respiratory distress. However, there are no standardized processes to assess for extubation readiness and marked variation among neonatal intensive care units (NICUs) persists [6]. Multiple strategies have been investigated, such as use of minute ventilation [7,8], spontaneous breathing tests [9], pulmonary and respiratory testing [10,11,12], cardiorespiratory variability [11,13,14,15,16], and diverse demographics and ventilator modes [17,18,19,20,21], all with variable success. The utility of the aforementioned predictors is inconsistent or of limited availability at the bedside. Consequently, an objective, feasible, and readily available assessment for extubation readiness is yet to be determined [22].

Pulse oximetry is commonly used in clinical practice, with adapted capabilities to calculate and display cumulative intermittent hypoxemia (IH) in the form of oxygen saturation (SpO_2_) histograms. Intermittent hypoxemia is likely underutilized in assessing extubation readiness, since IH is the result of both lung disease and respiratory instability in preterm infants [23,24,25,26]. In this communication, we explore the value of IH in extubation successes and failures in preterm infants.

## 2. Materials and Methods

Infants <30 weeks gestational age (GA) were prospectively enrolled upon admission to the neonatal intensive care unit (NICU). High-resolution SpO_2_ data were collected (sampling rate: 1 Hz, averaging time: 2 s) and archived as previously described [27]. Respiratory support and extubation data were retrospectively collected from the medical records. Data timestamps related to IH and respiratory support were well organized in a research database or medical records flow sheets, respectively. Informed consent was obtained prior to SpO_2_ data collection. The study was approved by the University of Kentucky’s Institutional Review Board.

IH is calculated as percent time spent with hypoxemia (SpO_2_ <80%) and number of events per day when SpO_2_ dropped to less than 80% for a 4–180 s duration [28,29]. Software created through Matlab was utilized to quantify IH measures [29]. IH measures were reported 24 h pre-extubation until 72 h post-extubation (or when reintubation became necessary).

At our institution, patients are considered for extubation when initial respiratory distress has improved and standardized readiness criteria are met (synchronized intermittent mandatory ventilation respiratory rate (RR) ≤20 breaths/min, fraction of inspired oxygen (FiO_2_) ≤40%, peak end expiratory pressure (PEEP) ≤6 cmH2O, peak inspiratory pressure (PIP) ≤20 cmH_2_O, and tidal volume (Vt) ≤6 mL/kg). All infants were extubated to non-invasive nasal support. We defined failure as reintubation within 72 h. Extubation events were grouped as successes or failures.

Statistical analyses were conducted in SAS Version 9.4 software (SAS Institute, Cary, NC, USA). Group comparisons of mean values for the percent time (%time IH- SpO_2_ <80) and the number of events (IH- SpO_2_ <80) were square root transformed, if necessary, in order to meet statistical assumptions as previously described [30]. Primary analyses defined failure as re-intubation before 72 h post-extubation. Receiver operating characteristic (ROC) curve analyses were used to find optimal cutoff values. Secondary analyses examined differences between groups at 24 h post-extubation. All tests were two-sided at the 5% significance level.

## 3. Results

Of the 91 extubations identified, a total of 68 extubation occurrences from 50 preterm infants <30 weeks of gestation had complete data sets and were therefore included. Demographics and respiratory support data are presented in Table 1. Median GAs were 26-6/7 weeks and 25-5/7 weeks in success and failure groups, respectively. All infants were extubated from conventional ventilator support to continuous positive airway pressure (CPAP) or noninvasive positive-pressure ventilation (NIPPV). Overall, most extubation attempts were successful (72% of events were successful at 72 h). As this was a pilot, we were not appropriately powered to detect group differences. Medians and interquartile-ranges are provided.

**Figure 1:**
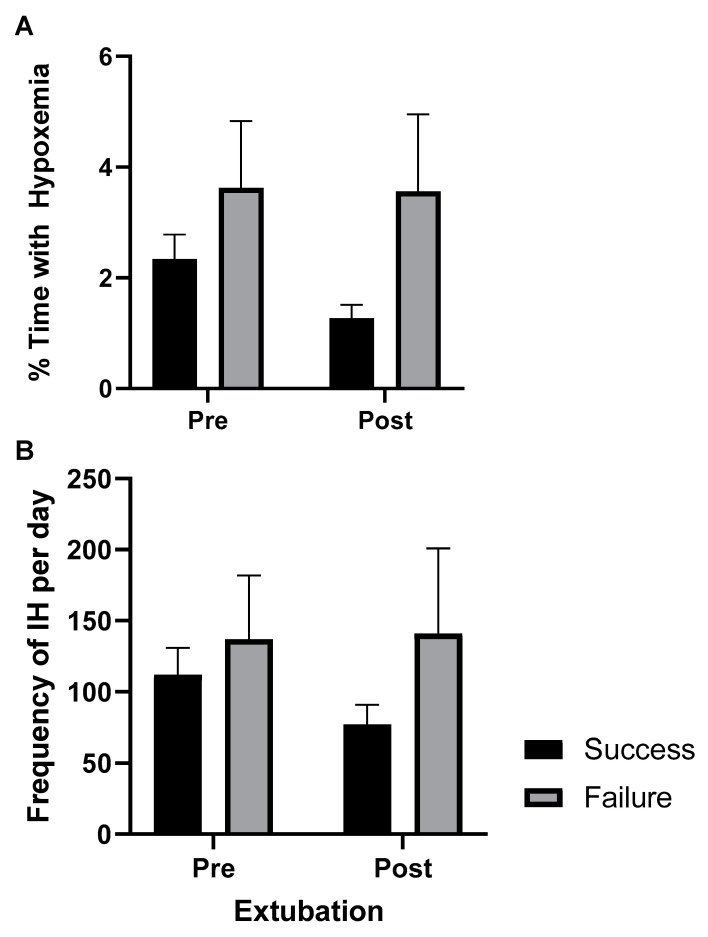
Intermittent hypoxemia (IH) measures 24 h pre- and up to 72 h post-extubation in success and failure groups. (**A**) Percent time in hypoxemia. (**B**) Frequency of IH. Mean ± SE.

Continuous SpO_2_ waveforms were interrogated for IH events and SpO_2_ histograms before and after extubation. Differences in IH measures between failure and success groups are represented in Figure 1; both %time- SpO_2_ <80 and IH- SpO_2_ <80 were higher in the failure group compared to successful group pre- and post-extubation, however, differences were not statistically significant. On the secondary analyses for differences in 24 h post-extubation, there were also increased IH measures in the failure group compared to the success group (%time- SpO_2_ <80, *p* = 0.07, and IH- SpO_2_ <80, *p* = 0.03). Interestingly, there was a statistically significant decrease in IH after extubation in the success group in both the primary (72 h) and secondary (24 h) analyses (all *p* < 0.01) (Figure 1). The differences in IH measures remained the same after adjusting for GA, birth weight, weight, and day of life at time of extubation. We applied the same analyses to the first extubation events only; these results were similar in IH trends.

Based on the ROC assessment, the optimal cutoffs of pre-extubation IH measures associated with successful extubation were calculated. The cutoff of 193 IH-SpO_2_ <80 per day and 7.5% with %time-SpO_2_ <80% had the highest sum of sensitivity and specificity. Example 1: if a patient had less than 193 IH events/day during the 24 h prior to extubation, there is a 73% likelihood of successful extubation for the patient. Example 2: if a patient who spent more than 7.5% of the time with SpO2 <80% per day were to be extubated, there is an 86% likelihood of failure for that patient.

## 4. Discussion

These pilot data demonstrate a potential and valuable role for IH in extubation success or failure in preterm infants. First, there was a trend for increased IH pre-extubation in the failure group as compared to the success group. Given that this was a pilot assessment with a limited sample size, these data support the need for a larger appropriately powered investigation of IH as a risk-predictor of extubation readiness. Second, the same trend between failure and success groups occurred post-extubation, indicating a potential role of IH in early detection of impending failures and prompt re-intubation. It has been reported that infants continue to have frequent IH events during mechanical ventilation [31]. Here, we document that infants who had successful extubations had a subsequent significant decrease in IH measures post-extubation. This interesting phenomenon may be useful for early identification of extubation success versus failure. IH may prove to be a reliable marker for both timely identification of extubation readiness and impending failure, lending to safe reinsertion of the endotracheal tube. Many studies have investigated extubation readiness in preterm infants [7,8,9,10,11,12,13,14,15,16,17,18,19,20,21]. Some studies utilized techniques such as pulmonary function testing that, although valuable, are not readily available at the bedside in every NICU. Other studies focused on ventilator settings and measures to assess for readiness. However, not all ventilators settings are weaned in a timely manner, and most may not provide accurate measurements of pulmonary mechanics. The value of IH, as derived from the SpO_2_ histogram, is both readily available in all NICUs and often an accurate consequence of a cardio-respiratory compromise. Limitations include the single-center retrospective nature of this study and limited power. In addition, while NICU criteria for extubation are in place, the final decision to extubate, or the need to reintubate remained at the discretion of the treating clinical team. Due to some subjects contributing multiple extubation observations, as well as the need to statistically account for repeated measures across time (pre- and post-extubation) from the same observations, multilevel modeling was employed, and thus we were under-powered to detect a significant difference. However, this is a novel study as the value of IH has not been examined in this setting.

Utilization of the bedside SpO_2_ histogram for cumulative IH as a predictor for extubation readiness may be a pragmatic answer for this decades-long problem of when is the optimal time to extubate infants recovering from respiratory distress [22]. Intermittent hypoxemia is a promising, objective, and feasible adjunct predictor for extubation readiness, as percent time with hypoxemia is readily available; often stored in the NICU bedside monitors for more than 24 h (SpO_2_ histogram). This is a pilot assessment, and we caution the readers from early implementation of the IH thresholds identified in this manuscript. We plan on a larger study to better define IH thresholds predictive of extubation success or failure in preterm infants.

## 5. Conclusions

Since IH is the result of respiratory instability and lung disease, we propose identifying cumulative IH (SpO_2_ histogram) as a valuable tool that guides clinicians’ decision making regarding extubation readiness or impending failure. A larger prospective study that utilizes IH as an adjunct predictor for extubation readiness is warranted.

## Figures and Tables

**Table 1:** Demographics and respiratory characteristics.

Baseline Characteristics	Success	Failure
	*n* = 49	*n* = 19
Gestational age (weeks)	26.6 (25.3–27.6)	25.5 (25.1–26.1)
Birth weight (grams)	890 (730–1040)	730 (650–905)
Weight at time of extubation (grams)	1140 (960–1253)	970 (830–1150)
Age at time of extubation (days)	18 (5–37)	21 (9–33)
Baseline Ventilator Setting		
• Set respiratory rate (breaths/min)	15 (15–20)	20 (15–20)
• FiO_2_ (%)	25 (21–30)	29 (25–32)
• PEEP (cmH_2_O)	6 (5–6)	6 (6–7)
• PIP (cmH_2_O)	15 (13–21)	16 (13–18)
• TV (mL/kg)	5 (5–6)	5 (4–6)
Post-extubation non-invasive support		
• CPAP	10/49 (20%)	0/19 (0%)
• NIPPV	39/49 (79%)	19/19 (100%)
• FiO_2_ (%)	32 (25–38)	40 (30–44)
• PEEP (cmH_2_O)	7 (6–8)	8 (7–9)

Median (interquartile range). FiO_2_: fraction of inspired oxygen, PEEP: positive end expiratory pressure, PIP: peak inspiratory pressure, TV: Tidal volume. CPAP: Continuous positive airway pressure. NIPPV: Noninvasive positive-pressure ventilation.

## Data Availability

The data presented in this study are available on request from the corresponding author.
